# Multi-omics characterization of a diet-induced obese model of non-alcoholic steatohepatitis

**DOI:** 10.1038/s41598-020-58059-7

**Published:** 2020-01-24

**Authors:** Helene M. Ægidius, Sanne S. Veidal, Michael Feigh, Philip Hallenborg, Michele Puglia, Tune H. Pers, Niels Vrang, Jacob Jelsing, Birgitte R. Kornum, Blagoy Blagoev, Kristoffer T. G. Rigbolt

**Affiliations:** 1 Gubra, Hørsholm Kongevej 11B, Hørsholm, Denmark; 2 Department of Biochemistry and Molecular Biology, University of Southern Denmark, Odense, Denmark; 3 Novo Nordisk Foundation Center for Basic Metabolic Research, University of Copenhagen, Copenhagen, Denmark; 4 Department of Neuroscience, University of Copenhagen, Copenhagen, Denmark

**Keywords:** Computational biology and bioinformatics, Drug discovery, Physiology, Systems biology, Pathogenesis, Hepatology, Endocrine system and metabolic diseases, Metabolic disorders

## Abstract

To improve the understanding of the complex biological processes underlying the development of non-alcoholic steatohepatitis (NASH), a multi-omics approach combining bulk RNA-sequencing based transcriptomics, quantitative proteomics and single-cell RNA-sequencing was used to characterize tissue biopsies from histologically validated diet-induced obese (DIO) NASH mice compared to chow-fed controls. Bulk RNA-sequencing and proteomics showed a clear distinction between phenotypes and a good correspondence between mRNA and protein level regulations, apart from specific regulatory events discovered by each technology. Transcriptomics-based gene set enrichment analysis revealed changes associated with key clinical manifestations of NASH, including impaired lipid metabolism, increased extracellular matrix formation/remodeling and pro-inflammatory responses, whereas proteomics-based gene set enrichment analysis pinpointed metabolic pathway perturbations. Integration with single-cell RNA-sequencing data identified key regulated cell types involved in development of NASH demonstrating the cellular heterogeneity and complexity of NASH pathogenesis.

## Introduction

Non-alcoholic fatty liver disease (NALFD) comprises a wide spectrum of liver diseases ranging from typically benign steatosis to non-alcoholic steatohepatitis (NASH), with or without fibrosis, that can progress into cirrhosis, hepatocellular carcinoma and ultimately end-stage liver disease^1–3^. The development of NASH is driven by complex and dynamic molecular mechanisms, implicating multiple parallel signalling pathways. However, the interplay between these different clinical and molecular manifestations linked to progression of NAFLD into NASH is not fully understood. Currently, no pharmacological therapies for NASH exist, however lifestyle modifications have shown to be efficacious for NASH resolution^4^. There are no early diagnostic endpoints known, and since hepatic steatosis and fibrosis can present itself as asymptomatic there is an unmet need to better understand the etiology and pathogenesis of NASH. Accordingly, several rodent models mimicking pathological features of NASH have been developed to accommodate this^5^. Due to the distinct hepatic features of NASH, histological techniques based on qualitative scoring systems^6,7^ and  quantitative image analysis have been developed for research applications. Omics-based strategies have been instrumental for hypothesis-free analysis of molecular changes in NASH. For example, genome-wide association studies have identified several loci with variants showing increased risk of NAFLD and NASH development^8–11^ or protection from more aggressive liver pathologies^12^. Furthermore, transcriptomics has been successful in identifying novel regulatory mechanism in NASH pathogenesis^13–17^. Despite the value of transcriptomics, these approaches will not always reflect the actual abundance at the protein level of a given the specific gene product in the cell^18^ or extracellular space/surrounding body fluids^19^. Quantitative proteomics has successfully been used for these purposes, uncovering biomarkers and hormones associated with NASH^16,17,20,21^. Recent advances in the field of mass spectrometry has allowed the utilization of proteomics in a more high-throughput setting with an increased depth of the analysis, further increasing the applicability of this technology for comprehensive comparative studies of global protein abundance across many experimental conditions, cell lines or tissues^22–24^. The development of new technologies providing full genome characterization of thousands of individual cells allowing transcriptomics analysis at the single-cell level has revolutionized the level of resolution and depth of physiological characterization attainable, resulting in a highly detailed profiling of cell types, often identifying hitherto unknown cell subtypes^25–27^.

To provide further insight into the complex molecular changes in fibrotic NASH, we applied a multi-omics approach, combining bulk RNA sequencing (RNA-seq) based transcriptomics, quantitative proteomics and single-cell RNA sequencing (scRNA-seq). The detailed characterization of the molecular signature of liver biopsies from diet-induced obese (DIO) NASH mice fed the AMLN diet expands our understanding of the numerous and intricate regulatory events underlying development of NASH.

## Results

### Histological validation of Diet-Induced Obesity mouse model of NASH

**Figure 1:**
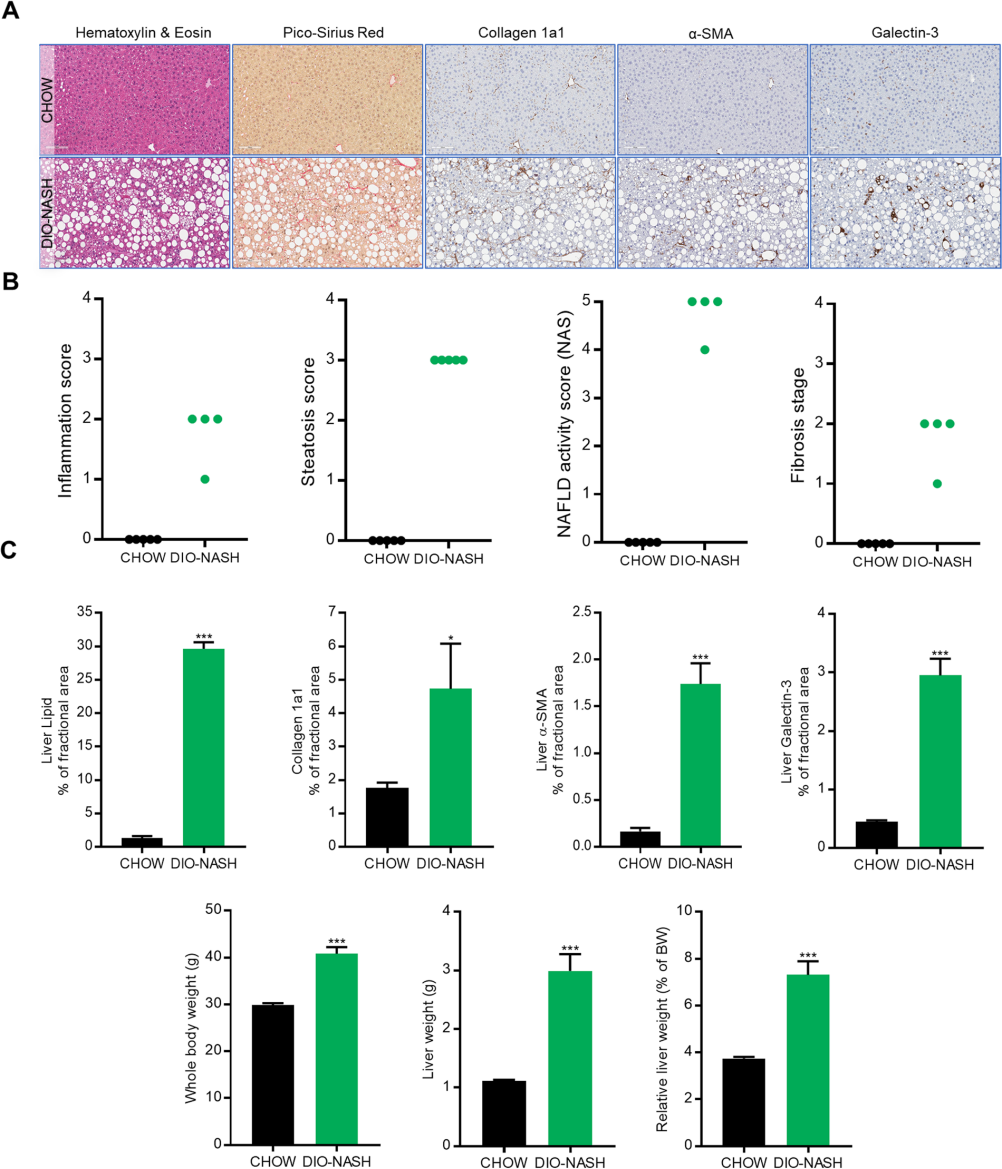
Histological characterization of DIO-NASH model – validation of NASH pathology. (**A**) Representative images of Hematoxylin & Eosin (H&E) and Pico-Sirius Red (PR) stainings, and immunohistochemical stainings of collagen 1a1, α-SMA and galectin-3. (**B**) Histological assessment of steatosis, inflammation and NALFD activity score (H&E) and fibrosis stage (PR). (**C**) Quantitative histochemical assessment of liver lipid content (p < 0.001) (H&E), collagen 1a1 (p = 0.042), α-SMA (p < 0.001), galactin-3 positive cells (p < 0.001) (% of fractional area), whole body weight (p < 0.001) and liver weight (p < 0.001). In panel C, data are expressed as the mean of n = 4-5 ± SEM. A student’s t-test was used. *p < 0.05, **p < 0.01, ***p < 0. 001 vs. CHOW.

Clinical hallmarks of NASH were evident after 36 weeks of ALMN diet feeding as revealed by increased steatosis and inflammation, but not hepatocyte ballooning (Fig. 1A,B). Scoring of fibrosis stage substantiated the fibrotic phenotype of DIO-NASH mice (Fig. 1B). Histopathological analyses were supported by quantitative image analysis of liver lipid, collagen 1a1, α-SMA, and galactin-3 content, all being significantly upregulated in the DIO NASH model versus chow control (*p* < 0.001, *p* = 0.042, *p* < 0.001, *p* < 0.001, Fig. 1C). DIO-NASH mice showed significantly increased whole body and liver weight (40.8 ± 1.435 g, 2.99 ± 0.29 g, n = 4) compared to chow controls (29.86 ± 0.403, 1.12 ± 0.02 g, *p* < 0.001, *p* < 0.001, n = 5, Fig. 1C).

### Characterization of changes in the liver transcriptome and proteome of DIO-NASH mice

**Figure 2:**
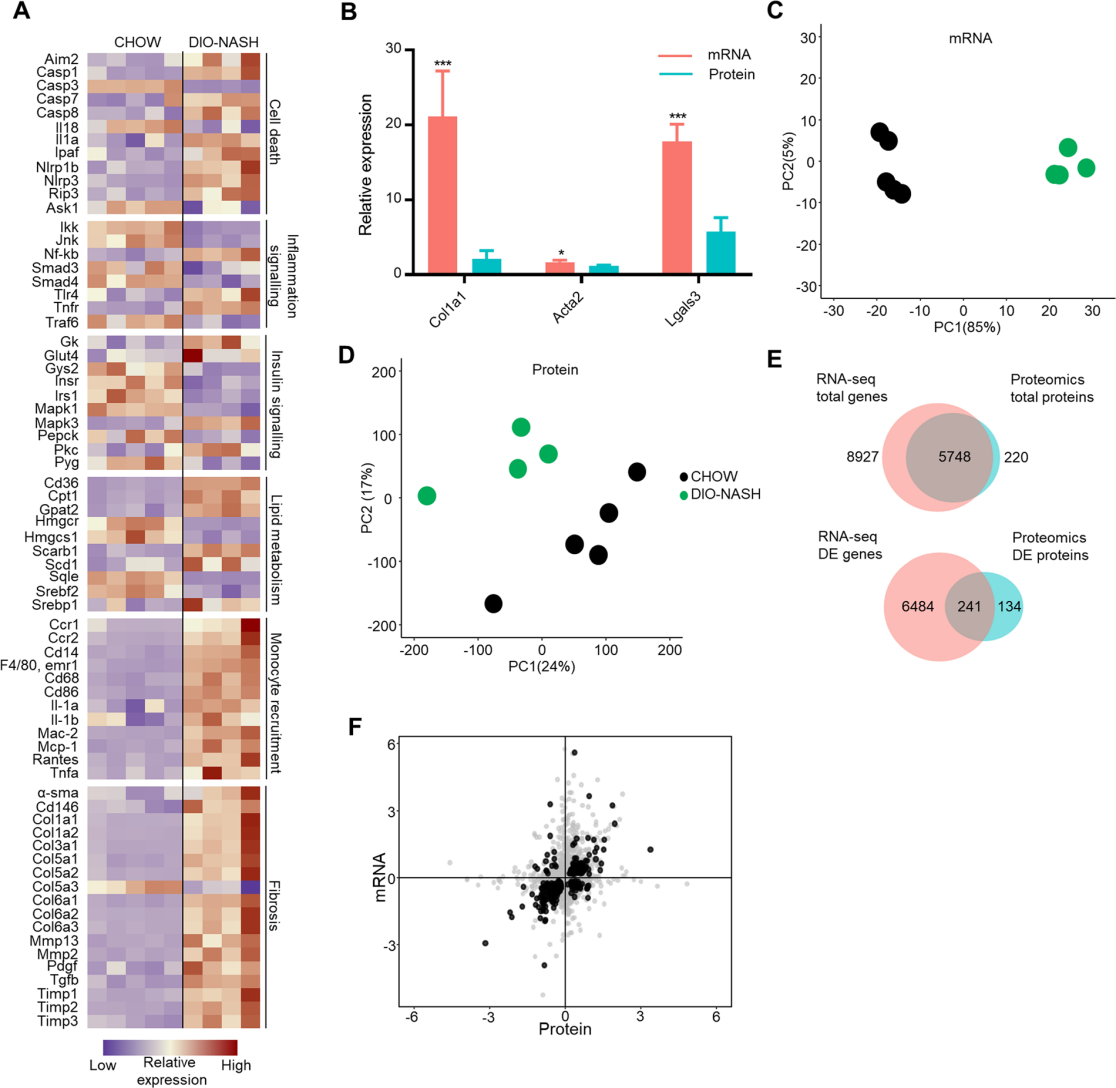
Model validation using transcriptomics and proteomics. (**A**) mRNA expression of genes (prototypical gene nomenclature) associated with NASH development in chow and DIO-NASH mice. (**B**) mRNA expression and protein abundance of histological markers Col1a1, α-SMA and Lgals3. (**C,D**) Principal component analysis (PCA) of the 500 most variable genes (**C**) and proteins (**D**). (**E**) Venn diagram showing total number of identified genes (red) and proteins (blue), the total amount of differentially expressed (DE) genes and proteins and the overlap between total number of identified and DE genes and proteins. (**F**) Correspondence between mRNA and protein expression. Black dots represent common differentially expressed genes and proteins (adjusted p < 0.05). In panel (A), data is presented as standardized relative expression. In panel (B), data is presented as fold change of mean of n = 4-5 ± SEM (DIO-NASH/CHOW) (relative expression). * adjusted p < 0.05, ** adjusted p < 0.01, *** adjusted p < 0. 001 vs. CHOW. In panel (F), data is presented as log2 fold change (DIO-NASH/CHOW). For all panels (A–F), n = 4-5.

Quantitative transcriptome and proteome analysis provided deep profiling of the molecular changes in the livers of DIO-NASH mice, identifying a total of 14,675 genes and 5,968 proteins. The hepatic transcriptome signature of DIO-NASH vs. chow-fed animals showed widespread regulation of genes with well-established association to NASH development with particularly large impact on prototypical fibrosis and monocyte recruitment associated genes^14^ (Fig. 2A). Quantitative proteomics and transcriptomics both showed increased expression of the standard NASH markers like Col1a1, Acta2 (α-SMA), and Lgals3 (Galactin-3/Mac2) in DIO-NASH compared to chow controls (transcriptomics: *p* < 0.001, *p* = 0.01, *p* < 0.001, Fig. 2B).

Principle component analysis (PCA) of RNA-seq data showed a clear distinction between groups reflecting the pronounced hepatic transcriptome changes in DIO-NASH mice vs. chow controls (Fig. 2C). Similarly, PCA of the proteomics data allowed for a separation of the mouse models, however, less distinct compared to mRNA (Fig. 2D). Of the 5,968 identified proteins, it was possible to match the majority to their corresponding gene in the RNA-seq dataset (Fig. 2E). Differential expression analysis identified 6,725 gene regulations and 375 protein regulations (*5% FDR*). Among the 375 regulated proteins, 241 were shared between the two datasets, while 134 were only identified as regulated in the proteomics data, suggesting widespread post-transcriptional regulation. Of the 241 shared regulations, 192 showed concordant directional changes at both protein and mRNA level. 14 genes were significantly increased at mRNA level but decreased at protein level, while 35 genes were significantly decreased at mRNA level but increased at protein level. Thus, a generally good correspondence between the mRNA expression and protein abundance profile was observed, albeit with clear indications of protein level specific regulatory events (Fig. 2F).

### Identification of physiological events globally regulated in DIO-NASH mice

**Figure 3:**
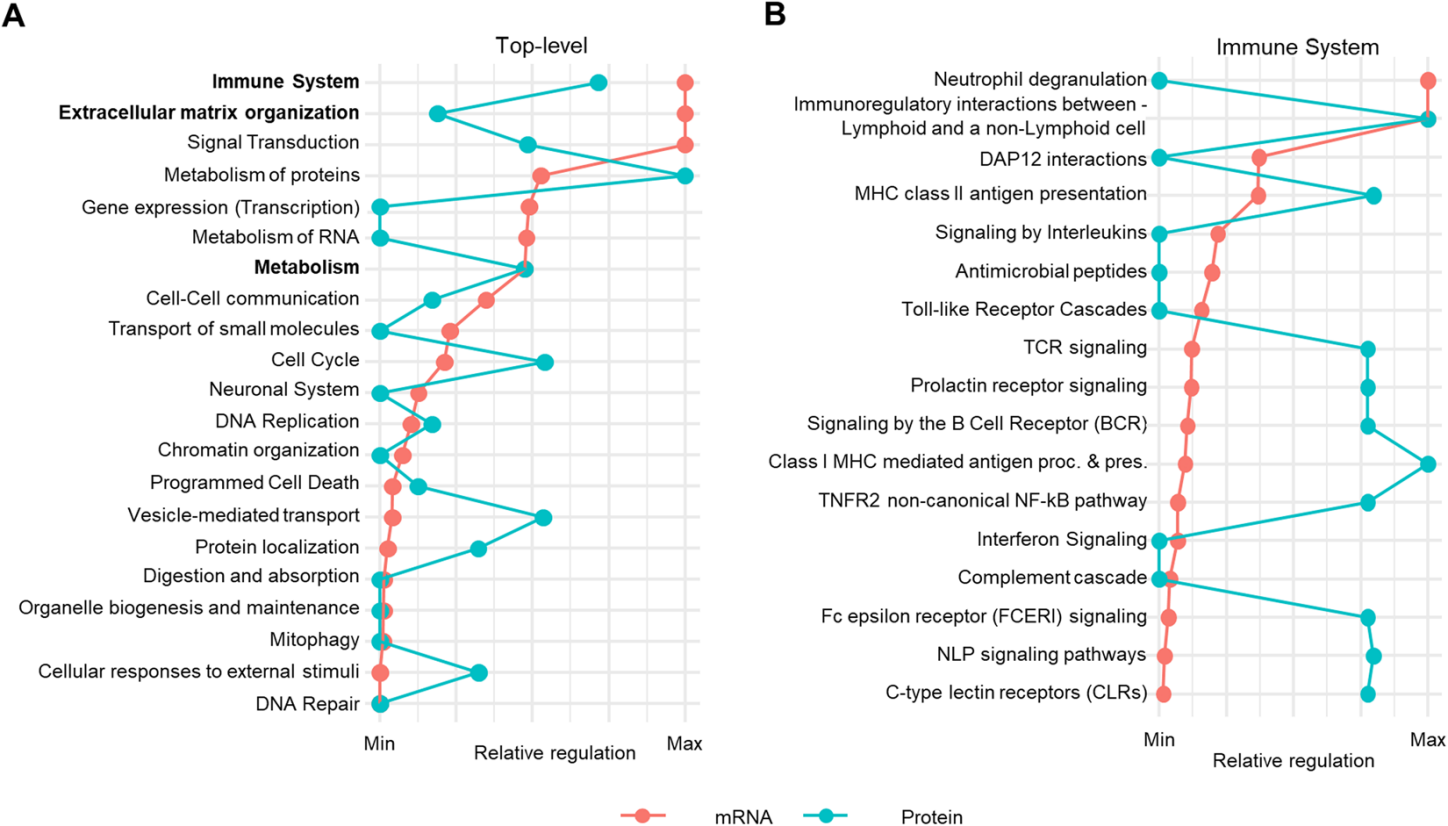
Global pathway analysis of transcriptomic and proteomic data. (**A**) Global transcriptome and proteome perturbations according to enrichment of individual gene sets in the Reactome pathway database indicating perturbed pathways in DIO-NASH vs. CHOW. (**B**) Transcriptome and proteome perturbations of *Immune system* sub-pathways. In panels (A,B), perturbed pathways are ranked according to level of statistical significance (mRNA only). The degree of perturbation for each pathway is normalized and presented as the relative regulation of the maximum p-value for each dataset.

The underlying physiological changes were explored further by gene set enrichment analysis. Comparable perturbations were observed for many top-level pathways (Fig. 3A). Pathways with well-established association to NASH pathogenesis were significantly perturbed on both mRNA and protein level, including *Immune System*, *Metabolism* and *Extracellular matrix organization*. Progression of NAFLD into NASH is associated with inflammation and liver cell damage. Notably, we observed an activation of all major aspects of the innate- and adaptive immune system, including upregulation of genes in pathways associated with the Toll-receptor Cascade, the Complement system, MHC II antigen presentation, T- and B-cell receptor activation and monocyte recruitment on mRNA level (see Fig. 3B). In contrast, protein regulations were more pathway specific with the strongest signal for processes associated with antigen processing.

**Figure 4:**
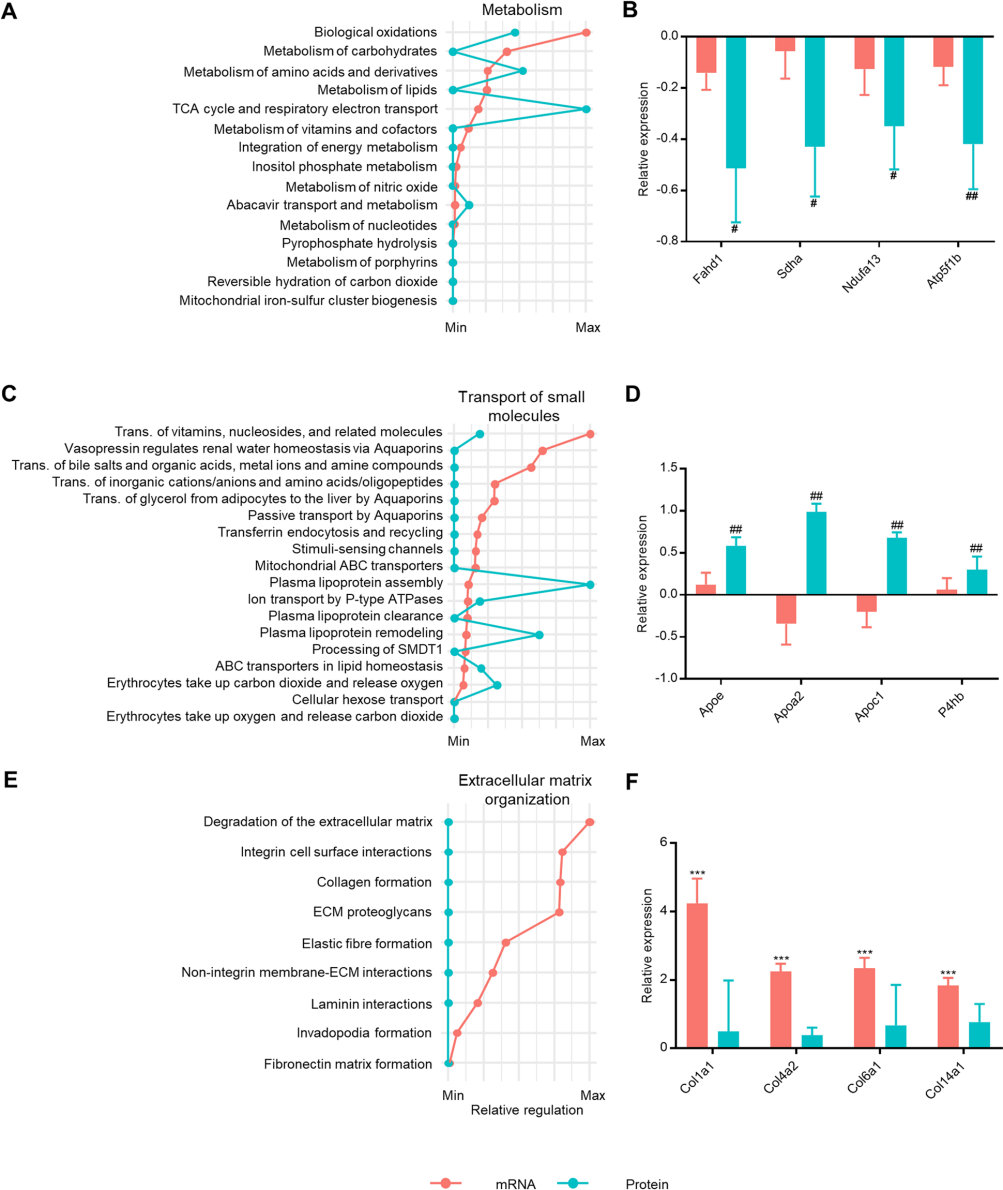
Metabolic and fibrotic pathway profiling. (**A**) Transcriptome and proteome perturbations of Metabolism sub-pathways. (**B**) mRNA expression and protein abundance of genes in the Metabolism pathway that are differentially expressed only on protein level. (**C**) Transcriptome and proteome perturbations of Transport of small molecules sub-pathways. (**D**) mRNA expression and protein abundance of genes in the Transport of small molecules pathway that are differentially expressed only on protein level. (**E**) Transcriptome and proteome perturbations of Extracellular matrix organisation sub-pathways. (**F**) mRNA and protein expression of genes in Extracellular matrix organisation pathway that are differentially expressed only on mRNA level. In panels (A,C,E), perturbed pathways are ranked according to level of statistical significance (mRNA only). The degree of perturbation for each pathway is normalized and presented as the relative regulation of the maximum p-value for each dataset. In panels (B,D,E), data is presented as the fold change of the mean of n = 4–5 ± SEM (DIO-NASH/CHOW) (relative expression). * adjusted p < 0.05, ** adjusted p < 0.01, *** adjusted p < 0. 001 vs. CHOW in RNAseq data. ^#^ adjusted p < 0.05, ^##^ adjusted p < 0.01, ^###^ adjusted p < 0. 001 vs. CHOW in proteomics data.

As the liver is a pivotal metabolic organ, alterations of hepatic metabolism are critical to the development of liver disease. Accordingly, gene and protein perturbations related to macromolecule metabolism were predominantly related to metabolism of lipids and carbohydrates (Fig. 4A). In this pathway, Fahd1, Sdha, Ndufa13 and Atp5b were differentially expressed only in the proteomics dataset (*p* = 0.03, *p* = 0.006, *p* = 0.04, *p* = 0,004, Fig. 4B). As lipoproteins are the main carrier by which lipids are distributed in the body, the metabolism of lipoproteins is believed to play a key role in dyslipidaemia observed in NASH^28,29^. Accordingly, lipoprotein assembly, remodelling and clearance pathways were significantly perturbed on both mRNA and protein level (Fig. 4C). However, several genes in this pathway were differentially expressed only in the proteomics dataset, including critical factors such as Apoe, Apoa2, Apoc1 and P4hb (*p* = 0.005, *p* = 0.004, *p* = 0.003, *p* = 0.003, Fig. 4D). Interestingly, only Apoa2 and Apoc1 were downregulated on mRNA level, with no change in the expression of Apoe and P4hb.

Hepatic fibrosis resulting from accumulation of extracellular matrix proteins produced by activated hepatic stellate cells plays an important role in disease progression of NASH^2,30^. Hence, the *Extracellular matrix organization* pathway was significantly upregulated on both mRNA and protein level, however, with relatively few differentially expressed proteins. Consequently, detailed analysis of *Extracellular matrix organization* associated pathways showed no significant perturbation on protein level, whereas high degree of perturbation was observed for all pathways on mRNA level (Fig. 4E). mRNA expression of collagen, a major constituent of extracellular matrix, was increased in DIO-NASH mice compared to chow control, while the increase on protein levels was not statistically significant (*p* < 0.001, *p* < 0.001, *p* < 0.001, *p* < 0.001, Fig. 4F).

Collectively, the pathway analysis validated the perturbation of NASH-associated pathways on mRNA level and furthermore identified several specific physiological processes that only could be detected by protein level analysis.

### Single-cell sequencing reveals cell specific regulations involved in NASH pathogenesis

**Figure 5:**
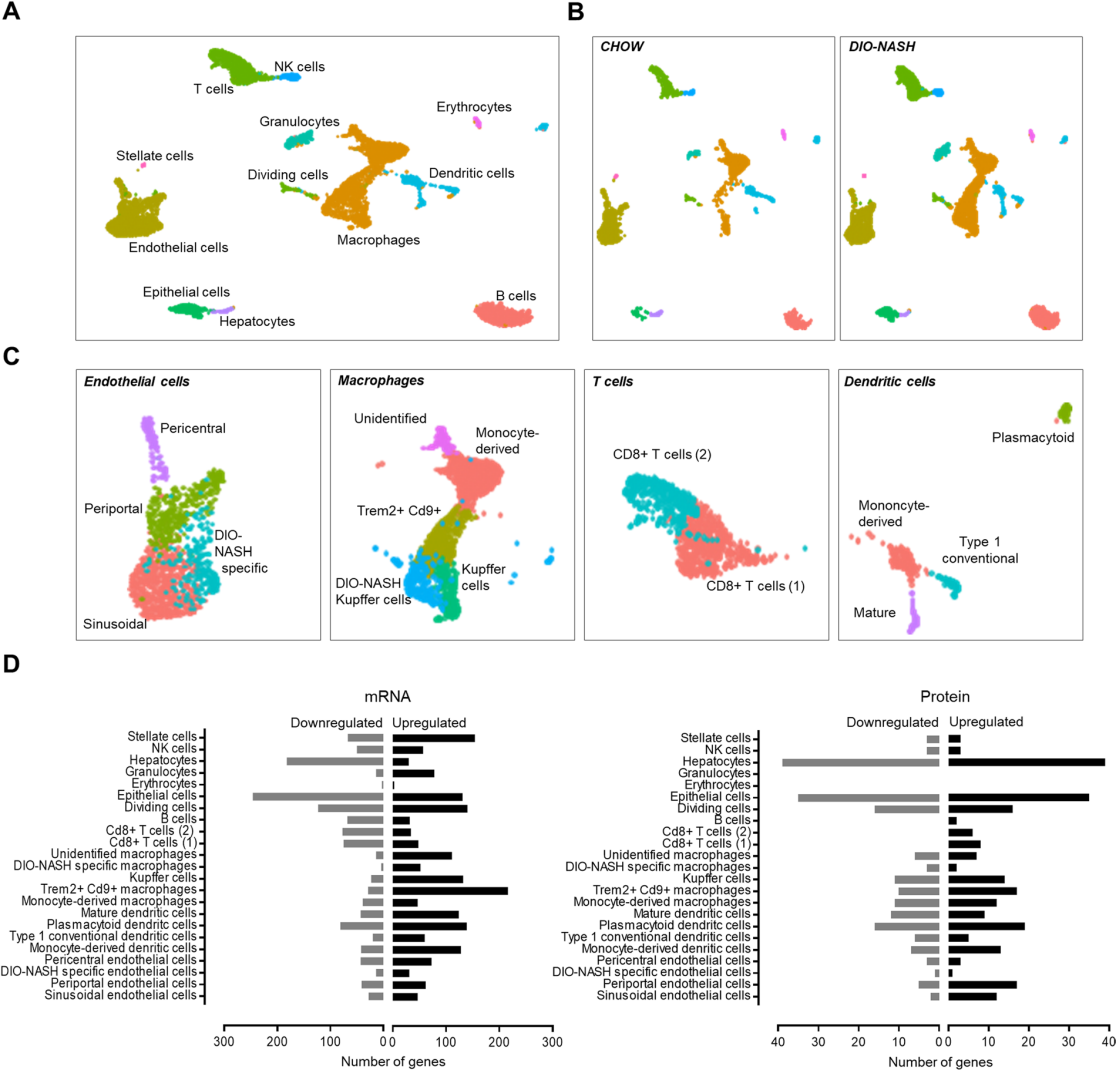
Discovering cell-specific changes in protein and gene levels of DIO-NASH mice. (**A**) UMAP projection of 6888 cells from CHOW and DIO-NASH livers. Each point represents a single cell. Cells that show similar transcriptomic profile are grouped by colour based on unsupervised clustering. 23 different cell populations were identified. Cell type identity was assigned by matching canonical markers with gene expression profile of each cluster. (**B**) UMAP projection showing how each experimental group contribute to each cell cluster. (**C**) UMAP projections showing sub-populations of endothelial cells, macrophages, T cells and dendritic cells. (**D**) Differentially expressed genes and proteins which was either up- or downregulated in DIO-NASH has been assigned to a specific cell-type as identified by scRNAseq by cross-referencing with marker genes (enriched genes).

To resolve the cellular heterogeneity of NASH development and physiological response identified by the pathway analysis, cell types involved in NASH pathogenesis were identified by single-cell transcriptomics. Using the 10x Genomics Chromium technology, the transcriptome of DIO-NASH and chow liver biopsies were profiled by scRNA-seq. All major cell types, including B cells, dendritic cells, dividing cells, endothelial cells, epithelial cells, erythrocytes, granulocytes, hepatocytes, NK cells, macrophages, stellate cells and T cells were identified in liver samples from both DIO-NASH mice and chow controls (Fig. 5A,B). Based on expression of subtype-specific marker genes, several cell types were resolved into subtypes, resulting in a total of 23 clusters corresponding to distinct cell populations. These included four endothelial subtypes, five macrophage subtypes, two T cell subtypes and four dendritic cell subtypes (Fig. 5C).

The four endothelial cells clusters are representing liver sinusoidal endothelial cells, periportal and pericentral endothelial cells and a population of unknown origin^31^. Liver sinusoidal endothelial cells showed enriched expression of Gpr182, Fcgr2b, Stab2 and Cd36. Periportal endothelial cells showed enriched expression of Tm4sf1, Efnb1, Id1 and F8. Pericentral endothelial cells showed enriched expression of Pecam1, Rspo3, Cdh13, Thbd and Wnt2. The last endothelial cell population showed high expression of Gpr128, Fcgr2b and Cd36 suggesting that these might also be sinusoidal cells. This cell population was almost exclusively found in NASH livers and showed increased expression of MHC class II molecules suggesting that these cells have an immunogenic expression profile. Five Cd68+ macrophage populations were identified with the largest being monocyte-derived macrophages, but also resident Kupffer cells were prevalent. One macrophage cluster had a transcriptomic profile resembling both monocytes-derived macrophages and Kupffer cells. These cells showed enriched expression of Trem2 and Cd9.

To investigate hepatic cell types associated with signalling pathway perturbations in DIO-NASH mice, subtype-specific marker genes from the single-cell expression atlas were cross-referenced to regulations from the proteomic and transcriptomic datasets. Based on the number of genes regulated, the major cell types perturbed in our DIO-NASH model were hepatocytes, epithelial cells, stellate cells, macrophages and sub-populations of T cells and dendritic cells (Fig. 5D). Interestingly, most of the hepatocyte and epithelial marker genes were downregulated in DIO-NASH, whereas most of the macrophage and stellate cell marker genes were upregulated in accordance with increased inflammation and fibrosis. Many stellate cell specific changes in mRNA level was observed supporting a highly perturbed *Extracellular matrix organization* pathway (Fig. 5D).

**Figure 6:**
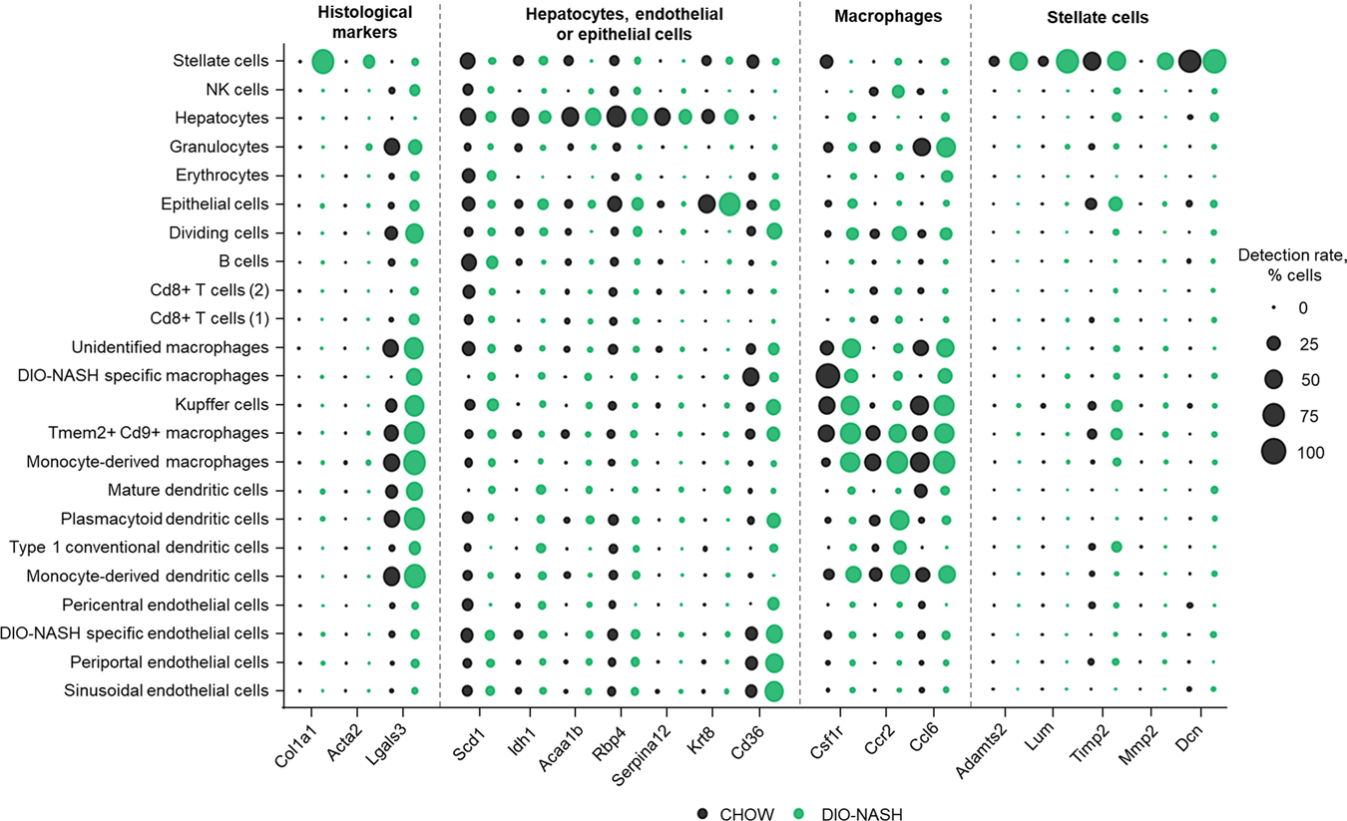
Uncovering regulated marker genes for key cell types involved in NASH pathogenesis. Cell specific distribution of gene expression of histological markers, Col1a1, Acta2 and Lgals3, and significantly regulated marker genes for hepatocytes, macrophages and stellate cells. Dot size represents the percentage of cells in a cluster expressing a given gene (detection rate, % cell).

To assign cell-type-specificity to regulations related to the histological manifestations of NASH, the distribution of gene expression of histological markers, Col1a1, Acta2 and Lgals3 was assessed (Fig. 6). Col1a1 and Acta2 expression were exclusively expressed in DIO-NASH stellate cells, whereas Lgals3 was expressed by most of the immune cell populations across both experimental groups.

To further characterize cell-specific regulations, significantly regulated marker genes for hepatocytes, macrophages and stellate cells were extracted, as they have been shown to be critical drivers of NASH pathogenesis (Fig. 6). In hepatocytes, gene markers were primarily associated with metabolic processes of the liver, especially lipid metabolism, as exemplified by a strong enrichment of Scd1, Idh1, Acaa1b, Rbp4, Serpina12 and Krt8, with the latter also showing enriched expression in epithelial cells. However, expression of lipid transporters, such as Cd36, were mainly present in endothelial cells and macrophages. Macrophages showed a clear enrichment of genes involved in cytokine signalling, albeit with several genes also showing less, but relevant, expression in other cell types. In contrast, the population of stellate cells was highly distinct with cell-type specific marker genes being essentially exclusively expressed in these cells, as evident for genes such as Adamts2, Lumican (Lum), Timp2, Mmp2 and Decorin (Dcn), all of which are involved in extracellular matrix production and remodelling. Overall, cross-referencing bulk RNA-seq and proteomics with scRNAseq allowed for assignment of major cell types to regulatory events and identified several key cell-specific regulations.

## Discussion

In the present study, we performed a comprehensive characterization of the molecular signature of a histologically validated diet-induced model of NASH. As previously reported^32–37^, the AMLN DIO-NASH model exhibits key hallmarks of NASH pathogenesis; steatosis, inflammation and moderate fibrosis. As in human liver pathologies, DIO-NASH mice are characterized by a large variation in disease progression and thus a careful validation of the phenotype is a prerequisite for the validity of omics data obtained from NASH animal cohorts. Histopathological scoring recapitulated clinical criteria for diagnosing fibrosing NASH^6,38^, albeit without ballooning degeneration. Quantitative histological assessment showed significant intrahepatic lipid accumulation, inflammation and collagen deposition in DIO-NASH compared to chow animals. We demonstrate widespread perturbations of the hepatic transcriptome and proteome, with overall good correspondence at the mRNA and protein level. Furthermore, combining bulk RNA-seq, quantitative proteomics and scRNA-seq constitute a powerful approach for dissecting the complex hepatic regulations associated with NASH. Notably, it provided us with a cell type specific pathway regulation atlas that augments the intrinsic complexity of NASH and heterogeneity of cell types involved in pathogenesis.

Differential expression analysis often assumes that changes in mRNA expression have biological meaning and induce corresponding changes in protein levels. However, moderate correlation between the proteome and transcriptome have been shown repeatedly^18,39^. Protein abundance is highly dynamic and multiple processes contribute to the abundance of a protein; mRNA abundance, translation efficiency, and protein turnover, all of which can be influenced by environmental conditions^40^. In addition, profiling the proteome has some inherent challenges; detecting low-abundance molecules can be difficult due to a wide dynamic range of protein expression, inefficient extraction of specific protein classes and difficulties with identifying small proteins. The present study demonstrate that transcriptomics analysis provides the most comprehensive dissection of the molecular signature of the DIO-NASH mouse model. However, as changes in mRNA level was not consistently accompanied by a change in protein level and vice versa, transcriptomic analysis alone would disregard important biological regulations. The explanation for the difference between identified regulations at mRNA and protein levels is likely multifactorial, with both biological and technological aspects contributing. Thus, a limitation of this study is the sample size that, combined with the variation in the animal model, could negatively impact the ability to identify differentially abundant proteins. The sample size could also influence the scRNAseq data preventing detection of extremely rare cells types, however, more influential is probably the fragility of hepatocytes leading to a loss of these cells during sample preparation.

Gene enrichment analysis revealed that proteome analysis was most efficient for identification of specific metabolic pathway perturbations, while RNA-seq was superior for characterizing regulations of immunological and fibrotic networks. Especially, the technical challenges associated with characterization of collagen proteins by mass spectrometry is worth noting since it has important clinical implications due to the slow turnover of many extracellular matrix proteins. We have previously shown that RNA-seq can efficiently detect reduction in expression of collagens by a panel of pharmaceutical compounds in advanced clinical trials, this is not mirrored in a histological reduction of liver fibrosis^37^. Other examples of the need for a multi-omics strategy is Rbp4 and Erlin1. Rbp4 is a carrier of retinol from liver into blood, which has received much attention as potential biomarker of NAFLD and NASH, however, the association is a matter of debate^41–45^. In this study, we found that the protein level of Rbp4 was significantly increased, whereas mRNA levels were significantly decreased in DIO-NASH mice. This is consistent with what previously have been found in a study including NASH patients where they showed increased serum Rbp4 levels^43^. Erlin1 is involved in regulation of cellular cholesterol homeostasis by regulation the SREBP signalling pathway^46^ and genetic variants of Erlin1 have been shown to be associated with increased susceptibility of hepatic steatosis and inflammation^11^. In this study, we found that the protein level of Erlin1 was significantly increased, whereas mRNA levels were significantly decreased in DIO-NASH mice.

The combined scRNA-seq and RNA-seq hepatocyte transcriptomic profile showed increased fatty acid synthesis and B-oxidation. Lipids not exported into the blood as VLDL particles form lipid droplets in hepatocytes, a defining feature of NASH^47^. Accordingly, the present gene enrichment analysis identified pathway perturbations associated with assembly, remodelling and clearance of lipoproteins suggesting an altered lipoprotein metabolism in DIO-NASH mice.

NASH has repeatedly been associated with mitochondrial dysfunction^48–52^ and the gene enrichment analysis showed impaired mitochondrial function by decreased expression of genes involved in TCA cycle, respiratory electron transport and ATP synthesis in DIO-NASH animals. Injured hepatocytes activate proinflammatory pathways by releasing cytokines, chemokines and other inflammatory mediators^53^. Consistently, hepatocyte specific expression of pro-inflammatory genes was upregulated in DIO-NASH and an overall upregulation of both the innate and adaptive immune system was observed.

Recently, Xiong and co-workers^16^ published a multi omics strategy to characterize a similar DIO-NASH model, albeit at an earlier timepoint and presumably less severe disease stage. In accordance to our dataset, Xiong *et al*. observe that most of the hepatocyte marker genes were downregulated in DIO-NASH, whereas most of the macrophage and stellate cell marker genes were upregulated, consistent with an upregulation of the *Extracellular matrix organization* and *Immune System* pathways. This data suggest that cell-type-specific reprogramming of the liver cell transcriptomes is linked to NASH pathogenesis.

Taken together, the molecular signature of the DIO-NASH model is associated with impaired intrahepatic lipid and carbohydrate handling, enhanced immune system activity and inflammatory signalling, mitochondrial dysfunction and enhanced extracellular matrix production and remodelling demonstrating the multifactorial nature of NASH. Hence, the AMLN DIO-NASH model demonstrates a high level of concordance with molecular and clinical manifestations of human NASH. Finally, the liver single-cell atlas revealed several key cell types involved in NASH, demonstrating the cellular heterogeneity and complexity of NASH pathogenesis.

## Methods

### Animals

All animal experiments were performed with ethical approval by the Gubra ethical committee and conducted in accordance with internationally accepted principles for the care and use of laboratory animals issued by the Danish Committee for Animal Research (license no. 2013-15-2934-00784, The Animal Experiments Inspectorate, Denmark).

Male C57Bl/6J mice at 5 weeks of age were obtained from Janvier Labs (Le Genest Saint Isle, France) and housed five per cage under a 12:12 hour dark–light cycle. Animals were either fed a diet high in fat/fructose/cholesterol (D09100301, Research Diets, New Brunswick, NJ, USA), previously described as AMLN-diet^33^ or regular rodent chow (Altromin 1324, Brogaarden, Denmark) and water. Both groups had ad libitum access to either the AMLN diet or rodent chow. After 36 weeks on diet animals were euthanized by heart puncture under 1-2% isoflurane anaesthesia and the left lateral lobe was collected for further analysis. 9 animals (DIO-NASH, n = 4, lean chow = 5) was used for proteomics and RNA-seq. 6 animals (DIO-NASH, n = 2, lean chow = 4) were used for scRNA-seq.

### Histology

Liver biopsies were collected from the left lateral lobe and fixed overnight in 4% paraformaldehyde. Liver biopsies were paraffin embedded, sectioned and stained with Hematoxylin & Eosin (Dako, Glostrup, Denmark), Pico-Sirius red (PSR, Sigma-Aldrich, Brøndby, Denmark), anti-type-1-collagen (Sourthern Biotech), anti-alfa-SMA (Abcam, Cambridge, UK) or anti-Galectin-3 (Biolegend, San Diego, CA) as described previously^36,37^. The NAS and fibrosis staging system outlined by Kleiner *et al*.^6^ was applied by experienced histopathologists to score liver biopsies for steatosis, lobular inflammation, hepatocyte ballooning, and fibrosis. Quantitative image analysis of stained liver sections were analyzed using the digital imaging software (Visiomorph; Visiopharm, Hørsholm, Denmark)^36,37^. Histo-chemical positive staining areas were expressed relative (%) to the total tissue sectional area (fractional area). Quantitative histological data were analyzed using GraphPad Prism v.7.04 software (GraphPad, LaJolla, CA), and results are shown as mean of n = 4–5 ± SEM. A students t-test was performed for quantitative histology data. All histological assessments were performed by a expert pathologist blinded to the experimental groups.

### RNA sequencing

Bulk liver transcriptome analysis was performed by RNA-seq of RNA extracts from liver biopsy samples (15 mg of fresh tissue). RNA sequencing libraries were prepared using the NEBNext Ultra II Directional RNA Library Prep Kit and sequenced (75 base-pair, single end reads) on a NextSeq 500 (Illumina, San Diego, CA, USA) with a NextSeq 500/550 High Output Kit V2 (Illumina, San Diego, CA). Reads were mapped to the GRCm38 v89 Ensembl *Mus musculus* genome using STAR v.2.5.2a (Dobin *et al*., 2013). The R-package DESeq. 2 v.1.18.1 was used for differential expression analysis (Love *et al*., 2014). P-values were adjusted using Benjamini-Hochberg method and genes with adjusted *p* < 0.05 was considered statistically significantly regulated. The Reactome pathway database^54^ was used as gene annotation for gene enrichment analysis using the R package PIANO v.1.18.1^55^, with the Stouffer method and Benjamini-Hochberg adjusted p-values.

### Proteomics Spectral library generation and Data Dependent Acquisition (DDA)

For quantitative comparison of the liver proteomes from CHOW and DIO-NASH mice, approximately 50 mg of liver tissue was homogenized and sonicated in denaturing buffer (8 M guanidine hydrochloride (GuHCl) in 25 mM ammonium bicarbonate). The protein samples were reduced with 2 mM DTT, alkylated with 11 mM chloracetamide, diluted with 25 mM ammonium bicarbonate to 2 M GuHCl and proteolytically digested with LysC (1:100) for 2 hours at room temperature. Following a dilution to 1 M GuHCl, the samples were digested with trypsin (1:100) overnight at room temperature. After acidification, peptides were desalted and lyophilized. A fraction (20 ug) of solubilized trypsin-digested peptides from all replica and condition were combined (200 ug total) and used to generate the spectral library for the following LC-MS/MS Data Independent Acquisition (DIA). The total peptide mixture was fractionated using high pH reverse phase chromatography on an Ultimate3000 HPLC system (Thermo Fisher Scientific) using a 10 cm long ACQUITY CSH C18 1.7 µm column (Waters) and a linear gradient of acetonitrile in water (pH 9) at a flow-rate of 5 µL/min for a total of 100 min. A total of 36 subfractions were collected and concatenated by combining subfraction #1 with #13 and #25, subfraction #2 with #14 and #26, etc. until obtaining a total of 12 fractions. The fractionated peptides, together with the input flow through (FT) fraction were processed as previously described^23^. Briefly, they were vacuum dried in a speed-vac and resolubilized in 7 μL of 0.5% acetic acid (AA) in water and used for the nanoLC-MS/MS analysis on a Q Exactive HF-X mass spectrometer coupled with an EASY-nLC 1000 ultra-high-pressure system (Thermo Fisher Scientific). Low pH reverse phase chromatography was performed on a 22 cm fused silica column with an inner diameter of 75 µm packed in house with C18 resin (1.9-µm beads, Reprosil, Dr. Maisch). Peptides were separated using a linear gradient of acetonitrile in water and 0.5% acetic acid (pH < 3) at a flow-rate of 0.25 µL/min for 110 min, followed by a washing phase of 10 min for a total of 120 min. The Q Exactive HF-X mass spectrometer was operated in positive polarity mode with spray voltage set to 2.3 kV and heated capillary temperature at 275 °C. MS data were acquired using a Data Dependent Acquisition (DDA) method switching between full scan events and the top 12 MS/MS scans. An automatic gain control (AGC) target value was set to 3 × 106 and resolution was set to 60,000 for full MS scan events with a scan range of 300–1,700 m/z and a maximum ion injection time (IT) of 15 ms. Precursors were fragmented by higher-energy collisional dissociation (HCD) with a normalized collisional energy (NCE) of 28%. MS/MS scans were acquired with a resolution of 30,000, maximum IT of 45 ms, 1.2 m/z isolation window. Repeat sequencing of peptides was prevented by setting the dynamic exclusion window to 60 s. The obtained Thermo raw files (12 HpH-RP fractions and 1 FT) were analyzed using MaxQuant software (Version 1.5.2.8)^56^. The MaxQuant search settings for maximum missed cleavages was set to 1, peptide mass tolerance to 4.5 ppm, fragment ion tolerance to 20 ppm and trypsin was chosen as enzyme. Variable modifications were specified to include oxidation on methionine and acetylation on protein N-term. As fixed modification, carbamidomethylation of cysteine was specified. MaxQuant data were filtered for reverse identifications, with FDR set as 1%.

#### Data Independent Acquisition (DIA) and data processing

After generating the spectral library using a pool of all mouse liver samples and a combination of high pH fractionation and DDA approach, the remaining trypsin-digested peptides from all samples were analyzed independently by LC-MS/MS using a Data Independent Acquisition (DIA) method. Briefly, while the nanoLC system (EASY-nLC 1000, Thermo) was operating exactly as described above for the DDA method, the Q Exactive HF-X mass spectrometer was set to analyze the samples as described previously^23^ with minor modifications. Spray voltage was set to 2.3 kV and funnel RF level at 50. Full MS mass range was set to 300–1,300 m/z and followed by 56 PRM spectra where the target masses have been adjusted to better fit the mouse spectral library compared to the human cell library used previously^23^. In this work the first target m/z was set to 319.5 until reaching 979.5 m/z as the last 56th target mass. Finally, normalized collision energy was set at 28%. DIA raw files were analyzed using Skyline v 4.2.0.18305 (MacCoss Lab software, University of Washington)^57^ using authors guidelines and settings. Library ion match tolerance was set to 5 mDa, MS/MS filtering was set to centroid with a 10ppm mass accuracy. Retention time filtering was set to use only scan within 10 min of library MS/MS identification time and to extract the Area Under the Curve (AUC) relative to the 5 most intense product ions for each peptide. mProphet peak scoring algorithm was trained against the decoy peptides library and used to identify correctly integrated target peptide with a Q-value < 0.01 (i.e. 1% FDR). Exported tables and subsequent analysis of data were performed in R. Differential expression analysis was performed by using two-way ANOVA on total sample intensity and group difference, with Benjamini and Hochberg corrections for multiple testing.

### Single-cell RNA sequencing

Single-cell liver transcriptome analysis was performed by scRNA-seq of liver biopsy samples (25 mg fresh tissue from left lateral lobe). Tissue biopsies were digested for 30 min at 37 °C at 200 RPM in Liberase DL (Roche, Basel). Following digestion, samples were kept on ice and the enzyme was quenched by adding PBS + 10% FBS. Samples were strained through a 70 µM filter and washed twice in PBS + 0.1% BSA. To remove red blood cells, samples were incubated for 5 min on ice with RBC lysis buffer (BioLegend, San Diego, CA). After incubation, samples were washed twice in PBS + 0.04% BSA and cell and viability count were assessed using a NucleoCounter NC-200. Samples were processed according to the 10x Genomics Single-cell 3′ v2 Reagent Kit user guide (10x Genomics, Pleasanton, CA). In brief, the volume of cells for specific target capture was determined for each sample. The appropriate number of cells were  loaded onto the single-cell-A-chip. Following GEM formation and barcoding, samples were transferred to Eppendorf tubes and reverse transcription was performed. Following reverse transcription, cDNA was recovered by using Recovery Agent (10x Genomics, Pleasanton, CA) and Silane DynaBead clean-up (Thermo Fisher, Waltham, MA). For cDNA amplification, the number of PCR cycles was chosen based on our target cell recovery. cDNA was amplified for 12 cycles and cleaned up using SPRIselect beads (Beckman, Brea, CA). Samples were run on a TapeStation (Agilent, Santa Clara, CA) to determine the cDNA concentration. Library construction was performed according to the user guide and the appropriate number of cycles for the sample index PCR reaction was chosen based on the cDNA concentrations. cDNA libraries were sequenced on a NextSeq 500 using NextSeq 500/550 High Output Kit v2.5 (150 cycles) (Illumina, San Diego, CA). Number of cycles were chosen based on 10x recommendations.

The sequencing data was aligned to the GRCm38 v89 Ensembl *Mus musculus* genome using the CellRanger (v. 1.1.0, 10x Genomics) software. Cell Ranger uses Spliced Transcripts Alignment to a Reference (STAR) software to align. For the bioinformatic analysis, the quality of the data was evaluated using standard RNA-seq quality control parameters. The CellRanger (10x Genomics, Pleasanton, CA) analysis pipeline was used to generate a digital gene expression matrix of the data. The Seurat R package (v. 3.1.1)^58^ was used to filter, normalize and cluster the data. Selecting a biological relevant number of clusters included performing PCA, determine differential expression between neighbouring clusters, creating a K-nearest neighbour graph and perform modularity optimization (Louvain algorithm). Differential expression between clusters was calculated using a likelihood-ratio test for single-cell gene expression implemented in Seurat at a family-wise error rate of 5%. Clusters were visualized using Uniform Manifold Approximation and Projection (UMAP). Cell identity was determined by examining the most enriched genes in each cluster (marker genes) and compare these with canonical marker genes of cell types known to be present in the liver.
